# Supplementary figures and images for: Theory and practice of using cell strainers to sort Caenorhabditis elegans by size
Source: PLoS One. 2023 Feb 9;18(2):e0280999. doi: 10.1371/journal.pone.0280999 (PMC9910635; doi:10.1371/journal.pone.0280999)

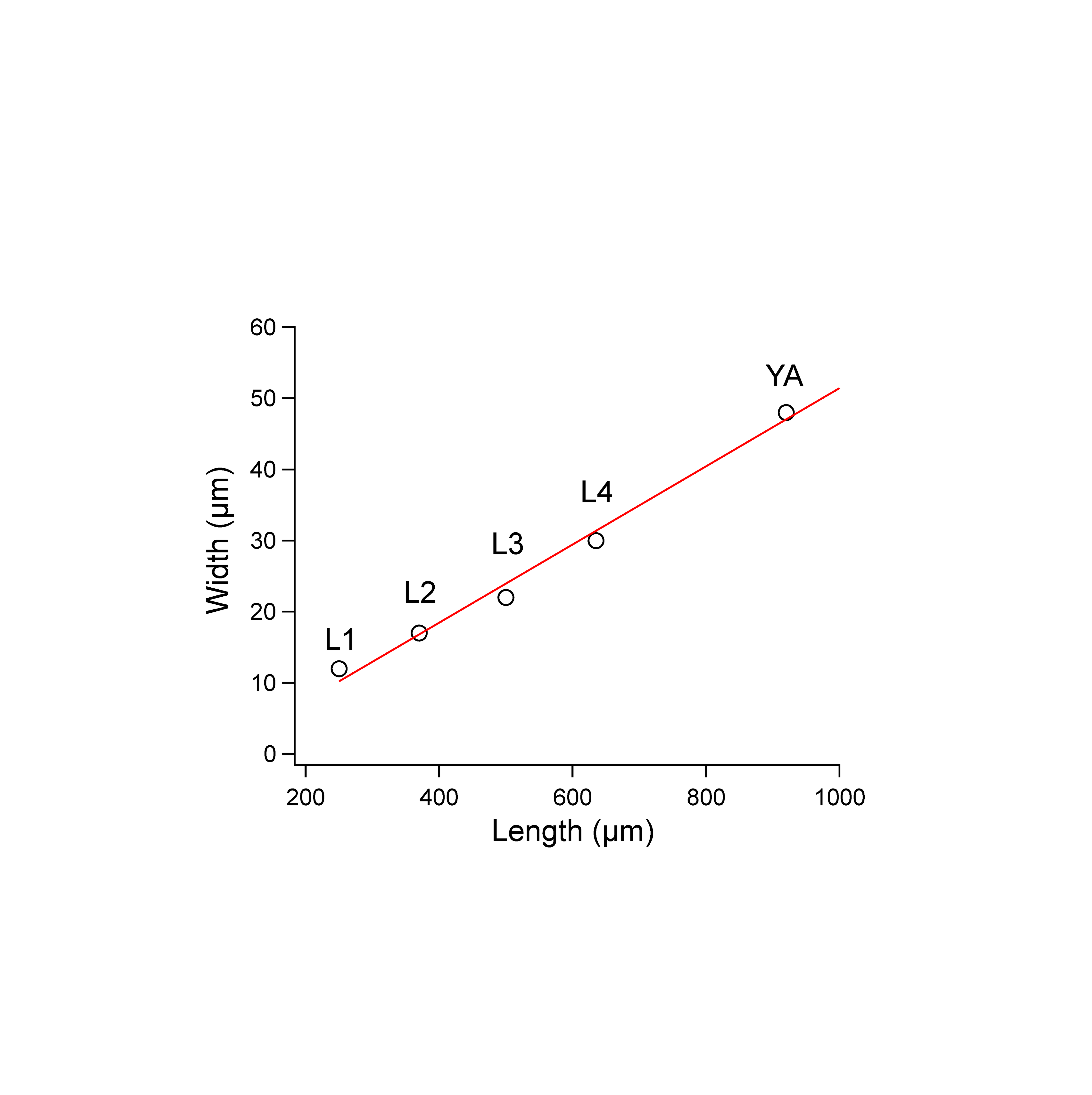

Supplement: S1 Fig — Widths of each stage were taken from measurements in Table 1 of Atakan et al. [7]. Lengths of each stage were the center of the length ranges that define each stage according to data in WormAtlas [23]. The data are fit by the equation w = 0.055l – 3.53, where w is width and l is length. (TIF) [file pone.0280999.s001.tif]

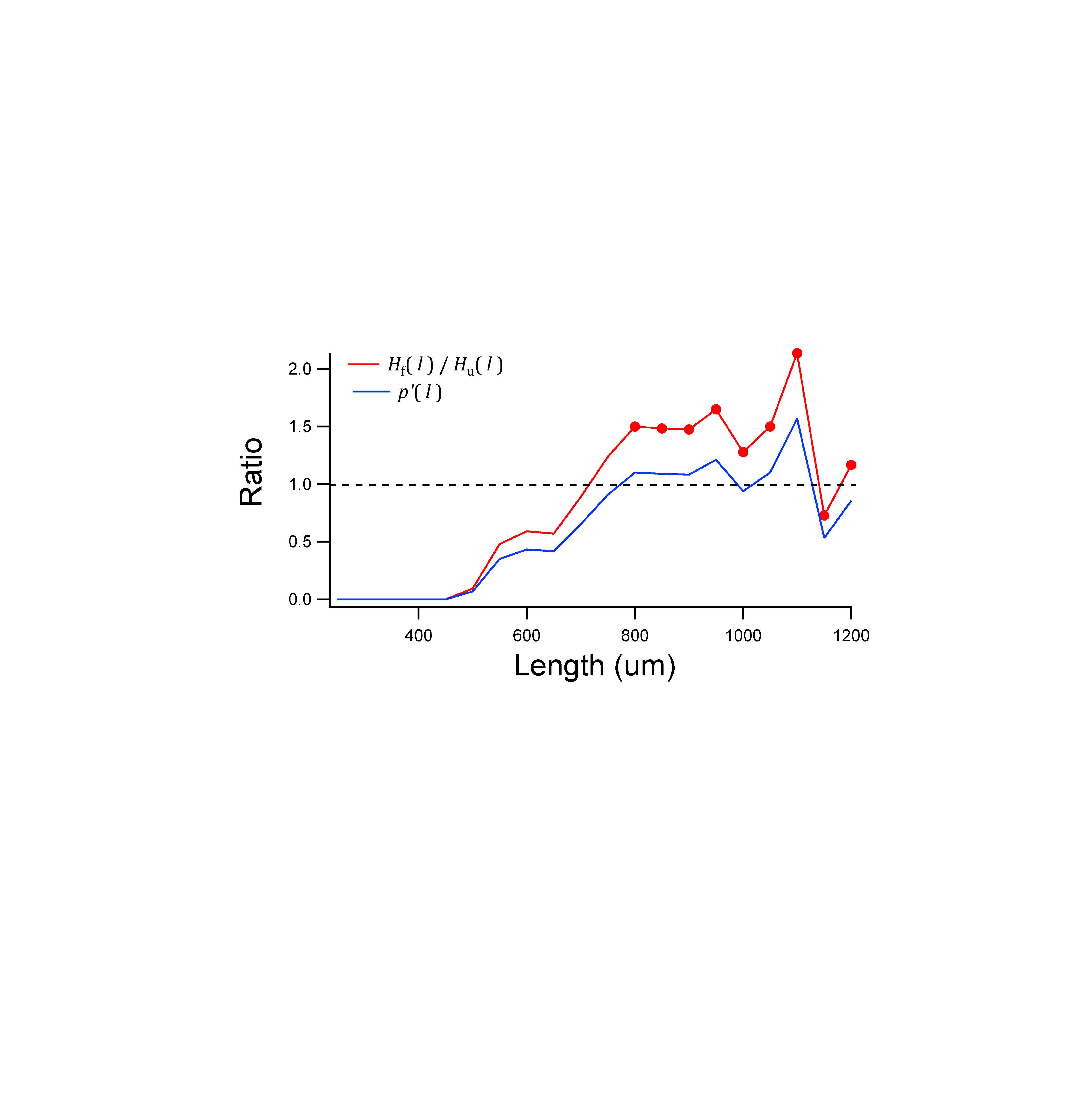

Supplement: S2 Fig — Red trace, the ratio Hf(l)/Hu(l) is plotted against length for one run of 30 μm retention. Blue trace, the ratio aHf(l)/Hu(l) = p’(l) for the same data (text Eqs 2 and 3), with a = 1/Xn and n = 9. The value of n was chosen by trial and error to optimize the fit of the logistic function (text Eq 4) to the mean of all runs in the 30 μm retention data set. (TIF) [file pone.0280999.s002.tif]
