# Supplementary material for: Theory and practice of using cell strainers to sort Caenorhabditis elegans by size
Source: PLoS One. 2023 Feb 9;18(2):e0280999. doi: 10.1371/journal.pone.0280999 (PMC9910635; doi:10.1371/journal.pone.0280999)
Supplement: S1 Table — Column n contains the number of values used to define the asymptotic region of the data during the fitting procedure. (DOCX) [file pone.0280999.s003.docx]

| **S1 Table**. **Filter-function parameters for fits to the data in the indicated figures.** | | | | | |
| --- | --- | --- | --- | --- | --- |
| **Filtration mode** | **Mesh size (μm)** | **Figure** | $\boldsymbol{l}_{\boldsymbol{o}}$ | $\boldsymbol{s}$ | $\boldsymbol{n}$ |
| Retained | 30 | 4A | 655 | 65.6 | 9 |
| Retained | 40 | 4B | 855 | 58.6 | 3 |
| Passed | 40 | 4C | 814 | 73.8 | 3 |
| Passed | 50 | 4D | 942 | 115 | 3 |
| Retained | 40 | 6C | 857 | 31.5 | 4 |
