# Supplementary material for: Theory and practice of using cell strainers to sort Caenorhabditis elegans by size
Source: PLoS One. 2023 Feb 9;18(2):e0280999. doi: 10.1371/journal.pone.0280999 (PMC9910635; doi:10.1371/journal.pone.0280999)
Supplement: S2 Table — aComponent Supply, Sparta, TN, 38583 USA. bpluriSelect, El Cajon, CA, 92020 USA. cFunakoshi, Tokyo, Japan. (DOCX) [file pone.0280999.s004.docx]

| **S2 Table. Sample of available nylon mesh sizes.** | | |
| --- | --- | --- |
| **Mesh fabric**  **(μm)^a^** | **Cell strainers (μm)^b^** | **Cell strainers (μm)^c^** |
| 7 | 1 | 25 |
| 10 | 5 | 40 |
| 15 | 10 | 70 |
| 18 | 15 |  |
| 25 | 20 |  |
| 31 | 30 |  |
| 38 | 40 |  |
| 40 | 50 |  |
| 44 | 60 |  |
| 52 | 70 |  |
| 56 | 85 |  |
| 60 | 100 |  |
| 62 |  |  |
| 64 |  |  |
| 70 |  |  |
| 80 |  |  |
| 85 |  |  |
| 105 |  |  |
|  | | |
