# Supplementary material for: Theory and practice of using cell strainers to sort Caenorhabditis elegans by size
Source: PLoS One. 2023 Feb 9;18(2):e0280999. doi: 10.1371/journal.pone.0280999 (PMC9910635; doi:10.1371/journal.pone.0280999)
Supplement: S1 Text — (DOCX) [file pone.0280999.s005.docx]

**Derivation of the logistic function**

Let $p(x)$ be the probability of a favorable outcome in a binary, categorical process, where $x$ is a continuous variable that predicts $p$. By definition, the odds of a favorable outcome are the ratio of the probability of a favorable outcome to the probability of a non-favorable outcome

| $O\left( x \right)=\frac{p(x)}{1-p(x)}$ | (1) |
| --- | --- |

In logistic regression, the parameters of a linear equation in $x$, such as $y = (x-\mu)/s$, are adjusted to fit the probability data $p(x)$ after transformation as log of the odds of a favorable outcome.

| $ln[O\left( x \right)]=(x-\mu)/s$ | (2) |
| --- | --- |

which is equivalent to

| $O\left( x \right)=e^{(x-\mu)/s}$ | (3) |
| --- | --- |

or

| $\frac{p(x)}{1-p(x)}=e^{(x-\mu)/s}$ | (4) |
| --- | --- |

Solving for $p(x)$ gives gives the familiar form of the logistic function

| $p(x)=\frac{1}{1+e^{-(x-\mu)/s}}$ | (5) |
| --- | --- |
